# Serum vitamin D concentrations are related to depression in young adult US population: the Third National Health and Nutrition Examination Survey

**DOI:** 10.1186/1755-7682-3-29

**Published:** 2010-11-11

**Authors:** Vijay Ganji, Cristiana Milone, Mildred M Cody, Frances McCarty, Yong T Wang

**Affiliations:** 1 Division of Nutrition, School of Health Professions, College of Health and Human Sciences, Georgia State University, 140 Decatur Street, Atlanta, GA 30302, USA; 2 Institute of Public Health, College of Health and Human Sciences, Georgia State University, 140 Decatur Street, Atlanta, GA 30302, USA; 3 Division of Physical Therapy, School of Health Professions, College of Health and Human Sciences, Georgia State University, 140 Decatur Street, Atlanta, GA 30302, USA

## Abstract

**Background:**

Vitamin D receptors have been mapped throughout the brain suggesting a role for vitamin D in psychosomatic disorders. Results from previous epidemiological studies on relation between vitamin D status and depression are equivocal. Also, limited information is available relating vitamin D status with depression in young adult US population.

**Methods:**

Data from the third National Health and Nutrition Examination Survey were used to assess association between serum vitamin D and depression in 7970 non-institutionalized US residents, aged 15-39 y. Assessment of depression was done using the Diagnostic Interview Schedule developed by the National Institute of Mental Health. After accounting for several confounding variables in multivariate logistic regression analysis, we estimated odds ratios (OR) for having depression in vitamin D deficient persons in comparison to vitamin D sufficient persons.

**Results:**

Women, non-Hispanic blacks, persons living below poverty, persons who did not consume supplements, persons living in South and West regions and in urban areas, persons with higher BMI, and persons with current depression had higher prevalence of vitamin D deficiency compared to their counterparts. OR for having current depressive episodes in persons with serum vitamin D ≤ 50 nmol/L is significantly higher relative to those with serum vitamin D ≥ 75 nmol/L (OR = 1.85; *P *= 0.021).

**Conclusions:**

In this large population based study, likelihood of having depression in persons with vitamin D deficiency is significantly higher compared to those with vitamin D sufficiency. Early diagnosis and intervention are paramount because coexistence of vitamin D deficiency and depression has serious negative consequences on health.

## Introduction

Low vitamin D status is a widespread problem in the US [1,2]. Research has shown that serum vitamin D concentrations previously considered in the normal range are not sufficient for optimal health [3]. Vitamin D plays a role in a wide range of ailments such as osteoporosis, cancer, cardiovascular diseases, and diabetes [4,5]. Recently, a role for vitamin D in cognitive function and mental health has been reported [6,7]. Vitamin D concentrations have been shown to be low in patients suffering from mood disorders and have been associated with cognitive function [8,9].

Depression is one of the leading causes of disability in the US among young adults. In the US, in a given year, about 26% of population, aged ≥18 years suffer from a diagnosable mental disorder and about 6% (1 in 17) suffer from a serious mental disorder [10]. Several mechanisms of action have been proposed to explain the association between vitamin D and depression. The role of calcitriol or 1, 25 dihydroxy cholecalciferol, the bioactive form of vitamin D, in brain tissue has been confirmed by the presence of vitamin D receptors (VDR) and hydroxylases in various brain regions [11,12]. One area where VDR and hydroxylases have been found is the amygdala, which is the center of the limbic system, where behavior and emotions are regulated [13]. Vitamin D has been reported to exert a neuroprotective function through several mechanisms. Calcitriol regulates calcium concentrations intra- and extracellularly in neurons, consequently reducing toxicity caused by excess calcium [14-16].

A few studies have found an association between serum vitamin D concentrations and depression [17-20]. Light therapy has been shown to improve the depression in adjunction with antidepressants, which may be in part due to improved vitamin D synthesis associated with light therapy [21]. Majority of the studies relating vitamin D status with depression are based either on a small sample size or non-representative of the US population. Very little is reported on the association between vitamin D concentrations and depression in young adult US population. Overall, the previous studies on the association between vitamin D status and depression yielded equivocal results [22-25]. Therefore, the aim of this study was to investigate the association between serum vitamin D concentrations (25 hydroxy cholecalciferol or calcidiol) and depression in a large, nationally representative sample survey of the US population, the Third National Health and Nutrition Examination Survey (NHANES III).

## Subjects and Methods

### Survey description

NHANES III was conducted by the National Center for Health Statistics (NCHS) of the Centers for Disease Control and Prevention (CDC) in 89 different geographic locations across the US, between the years of 1988 and 1994. Health and nutritional status of participants were collected using physical examinations, biochemical measurements of blood and urine samples, and health questionnaires. The physician administered physical examinations were performed in the mobile examination centers (MEC). All participants signed informed consent before participating in NHANES. A complete description of the survey procedures are described elsewhere [26].

Blood was collected by venipuncture at MECs, according to standard protocol. Centrifugation was used to separate serum after blood samples were held at room temperature for 30-60 minutes. Samples were sent to the CDC from MECs in a frozen state (-20°C) for biochemical analysis. Serum vitamin D concentrations were analyzed at the National Center for Environmental Health, CDC, Atlanta, GA using the DiaSorin RIA kit (Stillwater, MN) in participants >12 y of age [27].

### Study Sample

The current study sample initially consisted of 18875 participants whose serum vitamin D concentrations were measured. The diagnostic assessment for depression using the Diagnostic Interview Schedule (DIS) was collected from 8773 persons, aged 15-39 y. Participants with missing values for vitamin D concentrations, depression variables, and confounding variables were excluded from the analysis. Also, pregnant and lactating women were excluded from the study (*n *= 280). After applying the aforementioned exclusion criteria, the final study sample consisted of 7970 participants, representing over 90 million US non-institutionalized, civilian population (weighted *n *= 90759698). The mean (±SE) age of the study population was 27.5 (±0.16) y.

### Description of depression variables

The mental health component of the survey was collected in the MECs by trained interviewers using automated data entry, as part of the MEC Youth Questionnaire for examinees 15-16 y of age and as part of the MEC Adult Questionnaire for examinees 17-39 y of age. Depression was assessed using the DIS developed by the National Institute of Mental Health. The DIS is a complicated questionnaire with a number of parallel series of questions. The detailed description of the DIS questionnaire is described in Plan and Operation of the NHANES III [26,28]. The depression assessment derived from the DIS was used to make diagnoses based on the third edition of the Diagnostic and Statistical Manual of Mental Disorders (DSM III).

Below is the description of the DIS variables used in the data analysis.

1. SAS code, MQPDEP: DSMIII major depression

2. SAS code, MQPDYSA: >2 yrs of predominately depressive mood. Criteria for depression on the DSM-III are encompassed in this variable.

3. SAS code, MQPG35: Depressed spell now- "Are you in one of these spells of feeling low or disinterested and having some of these other problems now?"

### Description of confounding variables

Sex, age, race-ethnicity, geographical region, rural/urban setting, poverty income ratio, use of vitamin/mineral supplements and prescription medications, body mass index (BMI), and serum creatinine concentrations were used as potential confounding variables. These variables are spread over 4 databases (Household Adult File, Household Youth File, Examination File, and Laboratory File) [29-32]. Data were collected on geographical location (Northeast, Midwest, West, and South and urban/rural) of sampled persons. Persons race-ethnicity was classified into non-Hispanic white, non-Hispanic black, Mexican Americans/Hispanic, and Other categories. Individuals who reported taking vitamin/mineral supplements and prescription medications 30 day prior to the survey administered were classified as consumers of supplements and medications, respectively. Poverty income ratio was used to classify the person's socioeconomic status. Poverty income ratio is the ratio of a family's income to their appropriate threshold income. Persons with poverty income ratio <1.0, 1.0 to <2.0, 2.0 to <3.0, and ≥3.0 were considered as those below poverty line, low-middle income, high-middle income, and high income, respectively. BMI values <18.5, 18.5 to <25, 25 to <30, and ≥30 kg/m^2 ^were considered underweight, healthy weight, overweight, and obese, respectively.

### Data analysis

Statistical analysis was carried out with SUDAAN statistical software (SAS-callable version 10.0 Research Triangle Institute, Research Triangle Park, NC) and SAS (Statistical Analysis Software for Windows, version 9.1, Cary, NC). As per NHANES analytic guidelines, SUDAAN statistical software was used to account for NHANES's complex survey design. Variance estimates were computed using the Taylor Linearization Method assuming a design with replacement. Data were sorted by strata (SDPSTRA6) and Primary Sample Unit (SDPPSU6). Data were adjusted with the final MEC sample weight (WTPFEX6) in accordance to the NHANES III analytic and reporting guidelines [33]. Sample weights were used to correct for unequal probability of selection due to the oversampling of certain subgroups and to correct for non-response bias and differences between the sample and the total US population.

Although the vitamin D concentrations were not normally distributed, logarithmic transformation was not performed because in the analysis, serum vitamin D concentration was used as a categorical variable rather than a continuous variable. Serum vitamin D concentrations were divided into three categories, i.e., deficient (<50 nmol/L), insufficient (50-75 nmol/L), and sufficient (>75 nmol/L). The Chi-square test was used to find differences in the proportion of persons with vitamin D deficiency in various categories of the US population and in the participants with depression. Unadjusted and multivariate adjusted logistic regressions were used to generate the odds ratio (OR) and their 95% confidence intervals (CIs) for vitamin D concentrations and depression. In all analyses, the statistical significance was set at α = 0.05.

## Results

The study sample consisted of ≈54% women and ≈67% non-Hispanic black and Mexican American/Hispanics. Of 7970 participants, ≈30.6% lived in the Northeast and Midwest regions, ≈48% lived in rural areas, ≈27% lived below poverty, ≈23% used prescription medicine, ≈28% used vitamin/mineral supplements, ≈47% had BMI ≥ 25 kg/m^2^, and ≈4% reported that they had current depression at the time of the survey (Table 1).

**Table 1:** Characteristics of study population: the Third National Health and Nutrition Examination Survey¹

Characteristic	Value
Women	54.2
Non-Hispanic white	28.6
Non-Hispanic black	33.0
Live in South and West regions	70.4
Live in urban areas	51.9
Supplement use ^2^	28.4
Prescription medicine use ^3^	23.1
Persons living below poverty ^4^	27.2
Persons with overweight and obesity ^5^	47.2
Persons had a major depression	7.9
Persons had depression episodes longer >2 y	11.9
Persons having depression currently ^6^	4.0
Age (y)	27.5 ± 0.2
Poverty income ratio ^7^	2.74 ± 0.1
Body mass index (kg/m^2^)	25.3 ± 0.1

^1 ^*n *= 7970. Values are % or means ± standard error of mean

^2 ^Vitamin/mineral supplement use 1 month prior to the survey

^3 ^Prescription medicine use 1 month prior to the survey

^4 ^Poverty was defined as having poverty income ratio <1.0. Poverty income ratio is the ratio of a family's income to their appropriate threshold income.

^5 ^Overweight and obesity were defined as having BMI 25 to <30 kg/m^2 ^and ≥30 kg/m^2^, respectively

^6 ^Based on the question "Are you in one of these spells of feeling low or disinterested and having some of these other problems now"?

^7 ^The ratio of a family's income to their appropriate threshold income

Sample sizes and serum vitamin D concentrations for depression variables and confounding variables are reported in Table 2 and Table 3, respectively. The prevalences of vitamin D deficiency for depression and confounding variables are reported in Table 4 and Table 5, respectively. Higher prevalences of vitamin D deficiency were observed in women compared to men, in non-Hispanic black compared to non-Hispanic white and Mexican Americans/Hispanics, in persons below poverty compared to those above poverty, in persons who did not consume vitamin/mineral supplements compared to those who consumed supplements, in persons living in the South and in the West regions compared to those living in the Northeast and in the Midwest, in persons living in the urban setting compared to those living in the rural setting, in persons with higher BMI (≥25 kg/m^2^) compared to those with lower BMI (<25 kg/m^2^), in persons who had depression >2 y compared to those who did not have depression >2 y, and in persons with depression at the time of the survey compared to those who did not report having depression at the time of the survey.

**Table 2:** Serum vitamin D concentrations for depression variables of study population: the Third National Health and Nutrition Examination Survey¹

Characteristic	**Serum vitamin D **^**2**^**(nmol/L)**
Person had a major depression (*n *= 7877)	
Yes (*n *= 622)	79.8 ± 2.3
No (*n *= 7255)	77.9 ± 1.2
Persons had depression episodes longer >2 y (*n *= 7970)	
Yes (*n *= 948)	75.0 ± 1.8
No (*n *= 7022)	78.4 ± 1.2
Persons having depression currently (*n *= 1221)	
Yes (*n *= 322)	74.9 ± 2.7
No (*n *= 889)	81.2 ± 2.3

^1^Depression was assessed using the Diagnostic Interview Schedule (DIS) developed by the National Institute of Mental Health.

^2 ^Values are means ± standard error of mean

**Table 3:** Serum vitamin D concentrations for all confounding variables of study population: the Third National Health and Nutrition Examination Survey

Characteristic	**Serum vitamin D**^**1 **^**(nmol/L)**
Sex	
Men (*n *= 3652)	80.5 ± 1.3
Women (*n *= 4318)	75.7 ± 1.4
Race-ethnicity	
Non-Hispanic white (*n *= 2281)	86.5 ± 1.4
Non-Hispanic black (*n *= 2634)	48.5 ± 1.2
Mexican American/Hispanic (*n *= 2711)	65.3 ± 1.2
Other (*n *= 344)	63.0 ± 1.5
Geographical location	
Northeast (*n *= 958)	85.0 ± 2.3
Midwest (*n *= 1482)	86.3 ± 3.3
South (*n *= 3460)	71.8 ± 1.7
West (*n *= 2070)	72.6 ± 1.8
Urbanization	
Urban living (*n *= 4135)	72.5 ± 1.3
Rural living (*n *= 3795)	83.6 ± 2.0
Supplement use ^2^	
Yes (*n *= 2262)	81.0 ± 1.4
No (*n *= 4992)	75.9 ± 1.4
Prescription medicine use ^3^	
Yes (*n *= 1845)	79.3 ± 1.7
No (*n *= 5409)	77.2 ± 1.2
Poverty income ratio ^4^	
<1.0 (below poverty) (*n *= 2170)	69.9 ± 1.4
2.0-<3.0 (low-middle income) (*n *= 2058)	76.4 ± 1.7
3.0-<4.0 (high-middle income) (*n *= 2267)	79.3 ± 1.4
≥4.0 (high income) (*n *= 818)	84.5 ± 2.1
Body mass index (Kg/m2)	
Underweight (<18.5) (*n *= 277)	84.7 ± 3.5
Normal weight (18.5-<25) (*n *= 3911)	82.4 ± 1.3
Overweight (25-<30) (*n *= 2179)	76.3 ± 1.5
Obese (≥30) (*n *= 1583)	65.6 ± 1.5
Serum creatinine	
<70 μmol/L (*n *= 446)	70.2 ± 3.0
70-124 μmol/L (*n *= 7068)	78.6 ± 1.2
>124 μmol/L (*n *= 302)	72.2 ± 2.7

^1 ^Values are means ± standard error of mean

^2 ^Vitamin/mineral supplement use 1 month prior to the survey

^3 ^Prescription medicine use 1 month prior to the survey

^4 ^The ratio of a family's income to their appropriate threshold income

**Table 4:** Prevalence of vitamin D deficiency according to the depression status of study population: the Third National Health and Nutrition Examination Survey¹

Depression variables	Total (*n*)	Vitamin D **Deficiency **^**2**^ % (*n*)	Vitamin D **insufficiency **^**3**^ % (*n*)	Vitamin D **sufficiency **^**4**^ % (*n*)	**P-Value **^**5**^
Person had a major depression (*n *= 7877)					0.86
Yes	622	19.3 (215)	29.0 (191)	51.7 (216)	
No	7255	19.6 (2453)	30.6 (2458)	49.8 (2344)	
Person had depression longer >2 y (*n *= 7970)					0.039
Yes	948	24.7 (379)	29.6 (310)	45.7 (259)	
No	7022	19.1 (2322)	30.5 (2369)	50.4 (2331)	
Person having depression currently (*n *= 1221)					0.003
Yes	322	27.4 (138)	22.9 (99)	49.7 (95)	
No	889	14.5 (276)	32.6 (285)	52.9 (328)	

^1 ^Depression was assessed using the Diagnostic Interview Schedule developed by the National Institute of Mental Health.

^2 ^Serum concentrations of 25 (OH) vitamin D <50 nmol/L

^3 ^Serum concentrations of 25 (OH) vitamin D 50-75 nmol/L

^4 ^Serum concentrations of 25 (OH) vitamin D >75 nmol/L

^5 ^Significance of depression variable for proportions of persons with vitamin D deficiency, insufficiency, and sufficiency in the χ^2 ^test

**Table 5:** Prevalence of vitamin D deficiency in various categories of study population: the third National Health and Nutrition Examination Survey ^1^

Characteristic	**Deficiency **^**2**^ % (*n*)	**Insufficiency **^**3**^ % (*n*)	**P-value **^**4**^
All subjects	19.6 (2701)	30.4 (2679)	<0.001
Sex			<0.001
Men	15.5 (964)	31.1 (1304)	
Women	23.6 (1737)	29.8 (1375)	
Race-ethnicity			<0.001
Non-Hispanic white	10.1 (215)	28.5 (648)	
Non-Hispanic black	60.0 (1588)	28.7 (745)	
Mexican American/Hispanic	26.5 (790)	43.1 (1151)	
Other	32.4 (108)	38.0 (135)	
Geographical location			<0.001
Northeast	12.4 (288)	27.5 (284)	
Midwest	11.3 (391)	28.9 (493)	
South	27.1 (1467)	30.4 (1053)	
West	23.5 (555)	35.0 (849)	
Urbanization			<0.001
Urban living	25.1 (1612)	31.6 (1392)	
Rural living	14.2 (1089)	29.2 (1287)	
Supplement use ^5^			<0.001
Yes	15.8 (627)	29.5 (753)	
No	22.8 (1890)	30.1 (1657)	
Poverty income ratio ^6^			<0.001
<1.0 (below poverty line)	28.8 (849)	34.3 (797)	
2.0-<3.0 (low-middle income)	21.2 (745)	29.0 (672)	
3.0-<4.0 (high-middle income)	17.1 (692)	30.7 (725)	
≥4.0 (high income)	13.9 (192)	27.8 (242)	
Body mass index (kg/m^2^)			<0.001
Underweight (<18.5)	15.4 (71)	27.6 (98)	
Normal weight (18.5-<25)	16.0 (1128)	28.0 (1271)	
Overweight (25-<30)	20.3 (749)	31.9 (774)	
Obese (≥30)	31.0 (748)	36.3 (525)	

^1 ^Data associated with prescription medicine use and serum creatinine concentrations are not presented because these two variables were found non-significant in the χ^2 ^test for proportions of vitamin D deficiency, insufficiency, and sufficiency. Data associated with vitamin D sufficiency (>75 nmol/L) are not shown.

^2 ^Serum concentrations of 25 (OH) vitamin D <50 nmol/L

^3 ^Serum concentrations of 25 (OH) vitamin D 50-75 nmol/L

^4 ^Significance of variable for proportions of persons with vitamin D deficiency, insufficiency, and sufficiency in the χ^2 ^test

^5 ^Vitamin/mineral supplement use 1 month prior to the survey

^6 ^The ratio of a family's income to their appropriate threshold income

The OR and 95% confidence intervals for having depression and vitamin D deficiency are presented in Table 6. In unadjusted regression analysis, serum vitamin D concentrations were associated with depressive mood >2 y (OR: 1.43; P = 0.022) and current depression status (OR: 2.01; P < 0.001). When the analysis was adjusted for confounding variables (sex, age, gender, race-ethnicity, poverty income ratio, vitamin/mineral supplement and medication use, geographical location, rural/urban living, BMI, and serum creatinine) in the multivariate regression model, only the association between serum vitamin D and current depression status was significant (OR: 1.85; P = 0.021).

**Table 6:** Likelihood of having depression according to vitamin D status in study population: the Third National Health and Nutrition Examination Survey¹

Characteristic	Major depression OR (95% CI) (*n *= 7877)	Depression >2 y OR (95% CI) (*n *= 7970)	Current depression OR (95% CI) (*n *= 1221)
Unadjusted analysis for serum vitamin D			
Deficient (<50 nmol/L)	0.95 (0.65 - 1.37)	1.43 (1.09 - 1.86)	2.01 (1.25 - 3.24)
Insufficient (50-75 nmol/L)	0.92 (0.66 - 1.26)	1.07 (0.78 - 1.46)	0.75 (0.44 - 1.27)
Sufficient (>75 nmol/L) ^2^	1.0	1.0	1.0
P-Value ^3^	0.86	0.022	<0.001
Multivariate adjusted analysis for serum vitamin D ^4^			
Deficient (<50 nmol/L)	1.17 (0.71 - 1.90)	0.84 (0.57 - 1.23)	1.85 (0.90 - 3.81)
Insufficient (50-75 nmol/L)	0.93 (0.62 - 1.40)	0.77 (0.53 - 1.13)	0.70 (0.38 - 1.29)
Sufficient (>75 nmol/L) ^2^	1.0	1.0	1.0
P-Value ^3^	0.60	0.39	0.021

^1^Depression was assessed using the Diagnostic Interview Schedule developed by the National Institute of Mental Health. Odds ratios and 95% confidence intervals from the logistic regression analysis

^2 ^Referent category

^3 ^Significance for the Wald F in the multivariate logistic regression

^4 ^Logistic regression analysis was adjusted for sex, race-ethnicity, age, geographical location, urbanization, vitamin/mineral supplement use, prescription medicine use, poverty income ratio, body mass index, and serum creatinine

## Discussion

In this study, we investigated the association between vitamin D status and depression in the US population. Overall, the prevalence of suboptimal serum vitamin D concentration (≤75 nmol/L) was ≈50%. Out of 7970, ≈20% were vitamin D deficient (serum vitamin D <50 nmol/L) and ≈30% were moderately vitamin D deficient (serum vitamin D 50-75 nmol/L). Higher prevalence of vitamin D deficiency was found in women, in non-Hispanic blacks, in people with higher BMI, in people with lower income, in people living in urban areas, and in people living in the South compared to their counterparts.

These results are in agreement with the findings reported earlier [34,35]. What is surprising is that the prevalence of vitamin D deficiency was higher in persons living in the South compared to those living in the Northeast and Midwest given the more sun exposure in the South than in the North and that the direct sun exposure (UV light) leads to synthesis of endogenous vitamin D. This is likely due to the timing of the blood collection in the NHANES III. In order to avoid extreme cold climate in the Northeast and Midwest and warm climate in the South to prevent the damage to the MEC and to improve the participation, the NHANES III was conducted during summer in the Northeast and Midwest and during winter in the South (26). This could have significantly affected the prevalence of vitamin D deficiency in both these regions and more likely underestimated the prevalence of vitamin D deficiency in the North and overestimated the prevalence of vitamin D deficiency in the South.

A direct relation was observed between vitamin D deficiency and the depression variable "currently having depression episodes" in the multivariate adjusted regression model. This variable is the most relevant one, because this survey question was asked to specify the current depression status of the participants which coincides with the blood collection for vitamin D assay. In this study, participants with a current episode of depression had 8.4% lower concentrations of serum vitamin D compared to those who did not report having depression. This result is in accordance to previous epidemiologic studies [18-20]. In the Longitudinal Aging Study Amsterdam, Hoogendijk et al [20] found that serum vitamin D concentrations were 14% lower in persons with major and minor depressions and that the vitamin D status was associated with both major and minor depressions. Additionally, a randomized, double blind study in obese persons conducted by Jorde et al [19] reported that after 1 year of supplementation with vitamin D, subjects had significantly lower Beck Depression Inventory score and lower circulating parathyroid hormone without concomitant increase in calcium compared to those who received placebo. This study suggests a possible causal relationship between vitamin D status and depression.

In contrast to our findings, recently, Zhao et al [23] utilizing the data reported in NHANES 2005-2006 (*n *= 3916), found no significant association between serum concentrations of vitamin D and the presence of major depression, minor depression, and moderate to severe depression. However, they observed a trend of decreasing depression with increasing quartiles of serum vitamin D concentration in both unadjusted and multivariate adjusted regression models. Pan et al [22] also reported no significant association between vitamin D status and depression in Chinese adults aged, 50-70 y (*n *= 3262). It is very difficult to assess whether the differences observed between studies are due to true physiological differences or due to differences in methodology such as characteristics of study population, nutritional status of study participants, timing of the blood collection, assay used for the analysis of serum vitamin D, survey assessment used to collect the information on depression, and statistical adjustment used in regression models.

The mechanism through which vitamin D plays a role in metal health is not clearly understood. Active vitamin D enhances glutathione metabolism in neurons, therefore, promotes antioxidant activities that protect them from oxidative degenerative processes [16,17]. Vitamin D also stimulates the expression of nerve growth factor and promotes neuritogenesis [15,36]. Several studies have shown that vitamin D is involved in brain development and that its deficiency results in altered morphology (enlarged ventricles and reduced cortical thickness as it occurs in schizophrenia) and behavior in adulthood [37,38]. Moreover, it has been shown that vitamin D regulates gene expression of tyrosine hydroxylase, an essential enzyme involved in the synthesis of norepinephrine and dopamine [39]. Both neurotransmitters are involved in mood regulation and depression. Because vitamin D regulates calcium homeostasis, membrane permeability and axonal conduction, it is thought to have an indirect role in the regulation of neurotransmission.

## Conclusion

The likelihood of having depression in vitamin D deficient persons is significantly higher compared to those with adequate vitamin D status. A strength of this study is that the data were obtained from a nationally representative sample survey conducted on civilian residents, hence the results of this study can be applied to the population at large. Other strengths include the large sample size (*n *= 7970) and the use of several variables that influence serum vitamin D in the data analysis which allowed us to adjust the regression models for various confounding variables. Due to the cross-sectional nature of this study, the results should not be viewed in terms of cause and effect relationship.

It is not known, whether vitamin D deficiency leads to the depression or depression leads to the vitamin D deficiency. Further studies are needed in deciphering the precise role of vitamin D in psychosomatic disorders. Although the direction of the cause and effect relation between depression and vitamin D deficiency is not known clearly at this time, in public health perspective, the coexistence of vitamin D and depression in the US population at large is a concern. It is important to identify persons who are at risk for vitamin D deficiency and/or for depression and to intervene early because these two conditions have enormous negative consequences on long term health.

## Competing interests

The authors declare that they have no competing interests.

## Authors' contributions

VG, MMC, and CM designed the study. VG and CM wrote the manuscript. FM and YTW were responsible for the statistical analysis and data quality control. VG, CM, MMD, FM, and YTW are responsible for editing and revision of the manuscript. VG was responsible for the overall supervision of the project and is the corresponding author. All authors have read and approved the final content of the final manuscript.
